# Targeting Some Key Metalloproteinases by Nano-Naringenin and *Amphora coffeaeformis* as a Novel Strategy for Treatment of Osteoarthritis in Rats

**DOI:** 10.3390/ph16020260

**Published:** 2023-02-08

**Authors:** Nema S. Shaban, Abeer M. Radi, Mohamed A. Abdelgawad, Mohammed M. Ghoneim, Rasha Hamed Al-Serwi, Randa M. Hassan, Eman T. Mohammed, Rania A. Radi, Fatma M. Halfaya

**Affiliations:** 1 Department of Pharmacology, Faculty of Veterinary Medicine, Beni-Suef University, Beni-Suef 62511, Egypt; 2 Department of Pharmaceutical Chemistry, College of Pharmacy, Jouf University, Aljouf 72341, Saudi Arabia; 3 Department of Pharmacy Practice, College of Pharmacy, AlMaarefa University, Ad Diriyah 13713, Saudi Arabia; 4 Pharmacognosy and Medicinal Plants Department, Faculty of Pharmacy, Al-Azhar University, Cairo 11884, Egypt; 5 Department of Basic Dental Sciences, College of Dentistry, Princess Nourah bint Abdulrahman University, Riyadh 11671, Saudi Arabia; 6 Department of Cytology and Histology, Faculty of Veterinary Medicine, Beni-Suef University, Beni-Suef 62511, Egypt; 7 Department of Biochemistry and Chemistry of Nutrition, Faculty of Veterinary Medicine, Beni-Suef University, Beni-Suef 62511, Egypt; 8 Department of Surgery, Anesthesiology and Radiology, Faculty of Veterinary Medicine, Beni-Suef University, Beni-Suef 62511, Egypt

**Keywords:** *Amphora coffeaeformis*, nano-naringenin, metalloproteinases, rat MIA-osteoarthritis model

## Abstract

Osteoarthritis (OA) represents the highest degenerative disorder. Because cartilage erosion is a common pathological alteration in OA, targeting some key metalloproteinases such as MMP-3, ADAMTS-5 besides their inhibitor TIMP-3 by natural products, could be an effective strategy to protect against osteoarthritis. Forty female Wister rats were categorized into five equal groups. Control, osteoarthritic (OA) (monosodium iodoacetate (MIA) 2 mg/50 µL saline, single intra-articular injection), OA+ indomethacin (2 mg/kg/daily/orally), OA+ nano-naringenin (25 mg/kg/daily/orally), and OA+ *Amphora coffeaeformis* (772 mg/kg/daily/orally). Treatments were initiated on the 8th day after osteoarthritis induction and continued for 28 days thereafter. Finally, blood and knee joint samples were collected from all rats for biochemical and histopathological evaluations. The current study showed that MIA induced oxidative stress, which resulted in changes in the inflammatory joint markers associated with increased right knee diameter and higher clinical scores for lameness. *Amphora coffeaeformis* followed by nano-naringenin exhibited a potential anti-arthritic activity by reducing the concentrations of serum MMP-3, ADAMTS-5, and joint MDA and increasing the levels of serum TIMP-3 and joint GSH, similar to indomethacin. The histopathological results confirmed these outcomes. In conclusion, *Amphora coffeaeformis* and nano-naringenin can be considered as natural therapeutic agents for osteoarthritis owing to their antioxidant and anti-inflammatory activities.

## 1. Introduction

Over 10% of adults worldwide suffer from osteoarthritis (OA), which is among the most prevalent joint illnesses [1]. It is signified by several symptoms including joint pain, inflammation of, articular cartilage degeneration, osteophyte formation, and synovial joints [2]. During OA progression, extracellular matrix (ECM) macromolecules in the cartilage of the arthritic joint tissue are destroyed in great part by the increased activity of proteolytic enzymes [3]. In the cartilage of OA patients, there is a substantial degradation of aggrecan proteoglycans and type II collagen [4]. Disintegrin metalloproteinases with thrombospondin motifs (ADAMTSs) and matrix metalloproteinases (MMPs), which are associated with a metzincin superfamily of metalloproteinases and derived from both infiltrating and resident cells of the joint, are the primary proteinases responsible for the degradation of ECM components [5]. For example, aggrecanase 1 and 2 (ADAMTS-4 and ADAMTS-5) and MMP-3 are included in aggrecan breakdown and could be significantly blocked by TIMP-3. As a result, TIMP-3, which is generally found in cartilage, can prevent aggrecanases and MMPs directly, inhibiting aggrecan breakdown and therefore maintaining joint infrastructure [6]. 

A number of mechanisms, including increased chondrocyte death, pathologic cartilage matrix calcification, oxidative stress, increased chondrocyte cytokine-induced inflammation, and matrix catabolism have been linked to OA as a result of mitochondrial malfunction [7].

Nowadays, most anti-inflammatory medications, including indomethacin, disease-modifying anti-rheumatoid medications, including corticosteroids, hydroxychloroquine, leflunomide, sulfasalazine and methotrexate, including methylprednisolone and prednisolone, are effective at inhibiting inflammation, but they also have accompanied with dangerous adverse effects, including cardiovascular effects, gastrointestinal bleeding, and stomach ulcers. These side effects have motivated researchers to develop new pain therapies [8]. Natural products and their derivatives have been known as sources of new drugs in overcoming the existing adverse effects in pain treatment and various chronic diseases [9]. The upregulation of metalloproteinase inhibitors and suppression of proteolytic enzymes by phytochemicals can protect joints suffering from tissue damage.

The glycoside flavanone, naringenin (NG), found in citrus fruits and grapes, is the most common aglycone of naringin. NG has various medical applications including anti-diabetic, anti-dyslipidemia, antimicrobial, anti-obesity, and anticancer effects [10]. NG has been used for potent medical applications in osteoarthritis and rheumatoid arthritis due to its potential anti-inflammatory activities [11]. In comparison to bulky particles, nanoparticles have a higher surface area per mass unit and smaller size, which can lead to higher bioactivity and faster drug delivery [12]. As a result, the nano-size version of naringenin may be more effective than naringenin.

Due to the presence of a variety of beneficial phytoconstituents, marine algae display a diversity of biological activities [13]. *Amphora coffeaeformis* (AC) is a rich source of many pharmacologically active secondary metabolites such as vitamins C and E, α-tocopherol, polyunsaturated fatty acids, and flavonoids [14]. It contains a variety of photosynthetic pigments, including carotenoids (fucoxanthin and β-carotene), and chlorophyll, which have medical applications including antibacterial, anti-obesity, anti-cancer, and antioxidant properties [15], antiviral, and anti-inflammatory properties [16]. The *Amphora coffeaeformis* antioxidant activity is substantially more potent than that of α-tocopherol [17]. 

Little is known regarding the use of *Amphora coffeaeformis* and nano-size form of naringenin in osteoarthritic rats. Hence, this study has been planned to explore the possible potential anti-osteoarthritis activity of nano-naringenin as well as *Amphora coffeaeformis* extract in comparison with the most common anti-inflammatory drug; indomethacin against experimental MIA-osteoarthritis model in rats.

## 2. Results

### 2.1. Effect of Nano-Naringenin and Amphora Coffeaeformis on Body Weight

The changes in body weight in MIA-administered rats through the four weeks after induction of arthritis and treatment with indomethacin, nano-naringenin, and *Amphora coffeaeformis* were shown in Table 1. At zero days, there were no appreciable variations in body weight between different groups. After induction of arthritis in the first week, a significant (*p* ≤ 0.05) loss of weight gain in osteoarthritic animals was recorded, also osteoarthritic rats treated with indomethacin, nano-naringenin in comparison to the control group and osteoarthritic rats treated with *Amphora coffeaeformis*. Beginning on the second week, all treated rats gained more weight than MIA-administered rats over the duration of the test.

### 2.2. Effect of Nano-Naringenin and Amphora Coffeaeformis on Knee Swelling

Table 2 displays the progression of knee swelling in rats treated with indomethacin, nano-naringenin, and *Amphora coffeaeformis* over the course of four weeks following MIA injection. Significant swelling (*p* ≤ 0.05) in the right knee of osteoarthritic rats relative to non-treated rats was observed within the first week. The osteoarthritic control rats had a significantly larger right knee diameter than the normal rats up until the end of the experimental period. The osteoarthritic rats treated with indomethacin, nano-naringenin, and *Amphora coffeaeformis* produced a significant decline in the elevated level of the right knee.

### 2.3. Effect of Nano-Naringenin and Amphora Coffeaeformis on Clinical Score for Lameness

Mean values for lameness scores that were observed in normal, osteoarthritic, and treated groups during this study were shown in Table 3. There were four categories for lameness (mild, less moderate, moderate, and severe). Lameness was given clinical values of 1, 2, 3, and 4, correspondingly. The statistical averages of the recorded clinical lameness scores were assessed during the experiment. In the first and second weeks of the experiment, rats had the highest clinical scores for lameness. The treatment of the osteoarthritic rats with indomethacin, nano-naringenin, and *Amphora coffeaeformis* produced a significant decline in the elevated knee lameness score beginning in the third week compared to the osteoarthritic animals up until the completion of the investigation.

### 2.4. Effect of Nano-Naringenin and Amphora Coffeaeformis on Joint Redox Markers

The effect of indomethacin, nano-naringenin, and *Amphora coffeaeformis* treatments on joint oxidative stress (MDA) and antioxidant (GSH) markers of MIA-injected rats is represented in Table 4. The treatment of arthritic rats with indomethacin, nano-naringenin, and *Amphora coffeaeformis* produced a marked decrease in the elevated MDA. Hence, indomethacin, *Amphora coffeaeformis* followed by nano-naringenin seemed to be more potent in improving the MDA in osteoarthritic rats. Rats with osteoarthritis displayed a significant (*p* ≤ 0.05) decline in the joint GSH content in contrast to normal animals. As a result of *Amphora coffeaeformis*, indomethacin followed by nano-naringenin treatment, all of the deleterious effects of MIA on GSH levels were significantly (*p* ≤ 0.05) improved.

### 2.5. Effect of Nano-Naringenin and Amphora Coffeaeformis on Serum Pro-Inflammatory (ADAM TS-5, MMP-3) and Anti-Inflammatory Joint Markers (TIMP-3)

Data describing the effect of indomethacin, nano-naringenin, and *Amphora coffeaeformis* administration on the inflammatory joint markers (ADAM TS-5, MMP-3) and anti-inflammatory joint markers (TIMP-3) of MIA-administered rats are represented in Table 5. The results showed that MMP-3 and ADAM TS-5 increased sharply in MIA-administrated rats against the control group and treated groups. In contrast, TIMP-3 levels were elevated in normal control rats and treated groups compared to MIA-administrated rats. Hence, administration of indomethacin, *Amphora coffeaeformis* followed by nano-naringenin seemed to be more potent in improving the anti-inflammatory markers level and reduction of inflammatory joint markers.

### 2.6. Histopathological Results

#### 2.6.1. Effect on Histopathological Score

**Figure 1:**
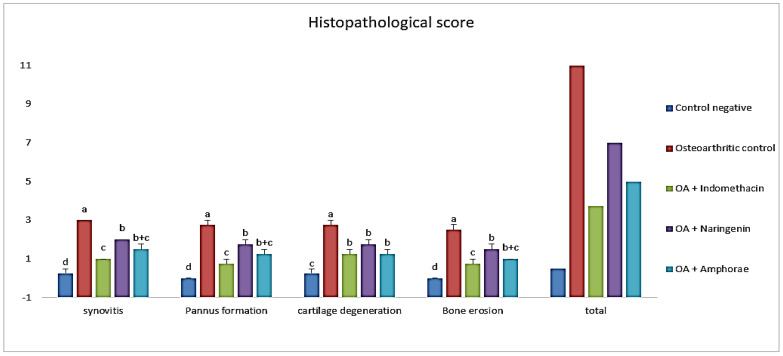
The scoring degree of arthritic lesions of all studied groups according to the (OARSI) scoring system. The different letters (a, b, c, d) indicate that the means are significantly different.

Osteoarthritis severity was evaluated in knee joint sections stained with H&E X40 by a blinded pathologist using the (OARSI) scoring system. The results of osteoarthritic lesions in all studied groups are compared in Figure 1. The current data showed that MIA-treated rats had a significantly (*p ≤* 0.05) lower overall histopathology score than osteoarthritic control rats.

#### 2.6.2. Histopathological Changes in Right Knee Joint

**Figure 2:**
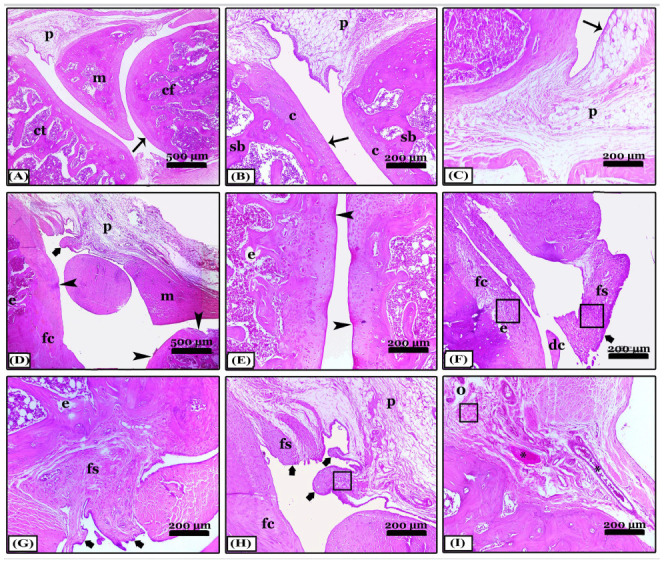
Photomicrographs revealed sagittal sections of knee joints appeared with H&E stain; (**A**–**C**) note; the control negative group showing normal; condyles of femur (cf) and tibia (ct), meniscus (m), periarticular tissue (p), spongy bone (sb), and regular articular cartilage with normal chondrocytes (c) and normal synovial membrane lining (thin arrows). (**D**–**I**) note; an osteoarthritic group with severe degenerative changes, articular cartilage degeneration (arrowheads), pannus formation of the synovial membrane (thick arrows), fibroid cartilage (fc), detached cartilage (dc), erosions (e), congestion (*), edema (o), fibroid synovial membrane (fs), inflammatory cell infiltrations (squares), meniscus (m), and periarticular tissues (p). All photomicrographs ×100 except (**A**) and (**D**) ×40.

**Figure 3:**
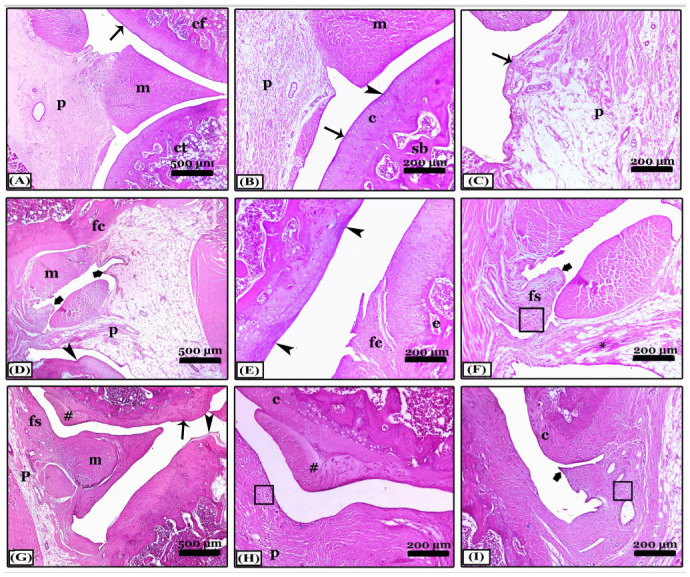
Sagittal sections photomicrographs of the knee joints appeared with H&E stain showing; (**A**–**C**) note; an indomethacin-treated group with normal condyles of femur (cf) and tibia (ct), meniscus (m), and periarticular tissues (p), minimal degenerative changes (arrowheads) in articular cartilages (c) with normal synovial membrane lining (thin arrows) and normal spongy bone (sb). (**D**–**F**) note; the nano-naringenin-treated group showed moderate inflammation. (**G**–**I**) note; *A. coffeaeformis*-treated group with minimal to mild inflammation. Notice, articular cartilage degeneration (arrowheads), pannus formation of the synovial membrane (thick arrows), fibroid cartilage (fc), erosions (e), congestion (*), fibroid synovial membrane (fs), regenerated cartilage (#), normal cartilage (c), periarticular tissues (p) and meniscus (m), and inflammatory cell infiltrations (squares). All photomicrographs ×100 except (**A**,**D**) and (**G**) ×40.

The difference between indomethacin, nano-naringenin, and *Amphora coffeaeformis* in the treatment of osteoarthritis was achieved in this study. The knee joint’s typical structure and integrity, including a normal synovial membrane, articular cartilages, and bones of femur and tibia condyles as well as menisci and periarticular tissue were seen in the sections stained with H&E from the control negative (Figure 2A–C). The osteoarthritic (OA) rats revealed severe degenerative and inflammatory changes: an indentation in the synovial membrane, multiple degenerated areas, irregularity of the articular surfaces with erosions in the cartilages which were replaced with granulation tissue, collagen fibers proliferation, and inflammatory cell infiltrations, in addition to chondrocyte apoptosis. Moreover, the articular bone erosions with numerous osteoclast activities (Figure 2D–F). Most OA rats have synovitis including periarticular vascular congestion, edema, inflammatory cell infiltrations, synovial lining cell proliferation, fibrosis, and extensive single and multiple pannus formation between the articular cartilages (Figure 2G–I). In contrast, most of the pathological lesions that showed in the osteoarthritic rats disappeared in the indomethacin-treated group except for minimal inflammation and fine degeneration of articular cartilage (Figure 3A–C). The severity of the lesions appeared less in osteoarthritic rats treated with nano-naringenin than in OA controls. There were multifocal moderate degenerative changes in articular cartilages, some replaced by collagen proliferation with inflammatory cell infiltrations, synovial tissue hyperplasia, and periarticular inflammatory cell infiltrations (Figure 3D–F). Although most of the lesions exhibited in the untreated group were markedly attenuated by the administration of *Amphorae coffeaeformis*, there were only minimal to mild degenerative changes in articular surfaces with cartilage regeneration in some areas, few periarticular inflammatory cell infiltrations, and minimal pannus formations (Figure 3G–I).

**Figure 4:**
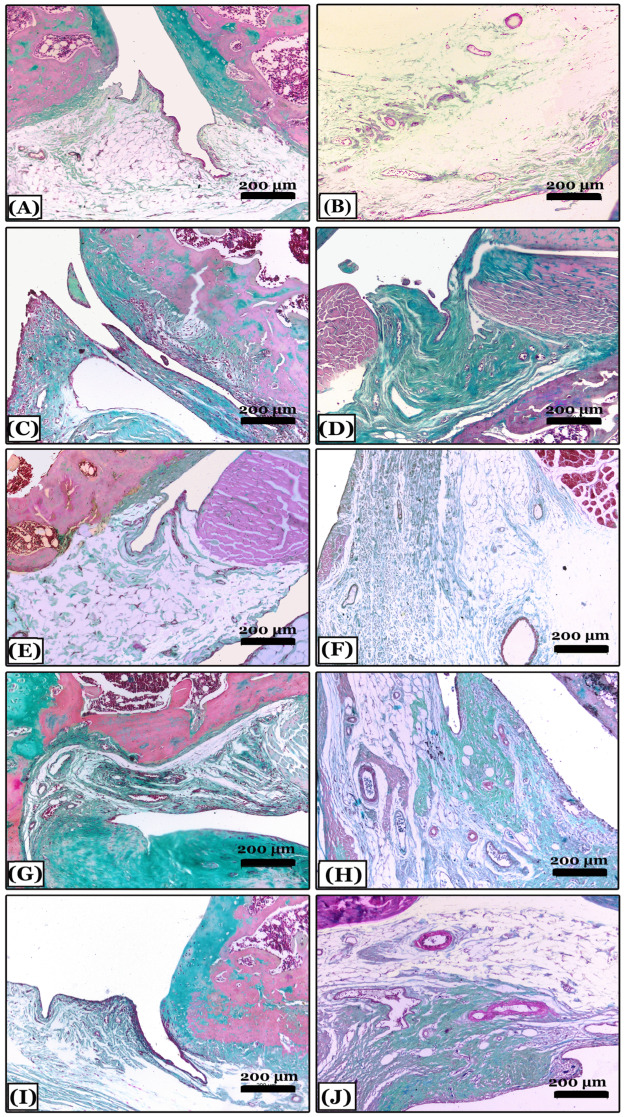
Histopathological evaluation of arthritic fibrosis in knee joints using Crossmon’s trichrome-stained sagittal sections from all studied groups. (**A**,**B**) note; the control negative group with a normal distribution of collagen fibers in the joint capsule. (**C**,**D**) note; an osteoarthritic group with a massive proliferation of collagen fibers in the synovial membrane forming multiple panni and the degenerated articular cartilage replaced with fibers. (**E**,**F**) note; an indomethacin-treated group with fine collagen fibers similar to normal control. (**G**,**H**) note; the nano-naringenin-treated group showed moderate collagen proliferation in the joint capsule. (**I**,**J**) note; an *Amphora coffeaeformis*-treated group with mild collagen proliferation. All photomicrographs ×100.

Regarding Crossman’s Trichrome stain, joint sections from the control negative revealed a typical collagen fibers distribution within the synovial membrane, periarticular tissue, or joint capsule (Figure 4A,B). In contrast, sections of the OA group appeared with a massive proliferation of collagen fibers in the synovial membrane and periarticular tissue, in addition to collagen fibers that replace the degenerated articular cartilage in different areas forming multiple panni between them (Figure 4C,D). Treatment with indomethacin prevented the fibrosed picture of arthritis, so, there were fine collagen fibers similar to normal control and cartilages were intact (Figure 4E,F). While the treatment with nano-naringenin improved the joint condition, which showed moderate collagen proliferation in the synovial and periarticular tissue, a small area of articular cartilage was replaced with fibers in comparison with the OA group (Figure 4G,H). However, in the *Amphorae coffeaeformis* treated group, sections revealed mild collagen proliferation in the joint capsule only without any cartilage replacement with collagen fibers (Figure 4I,J).

**Figure 5:**
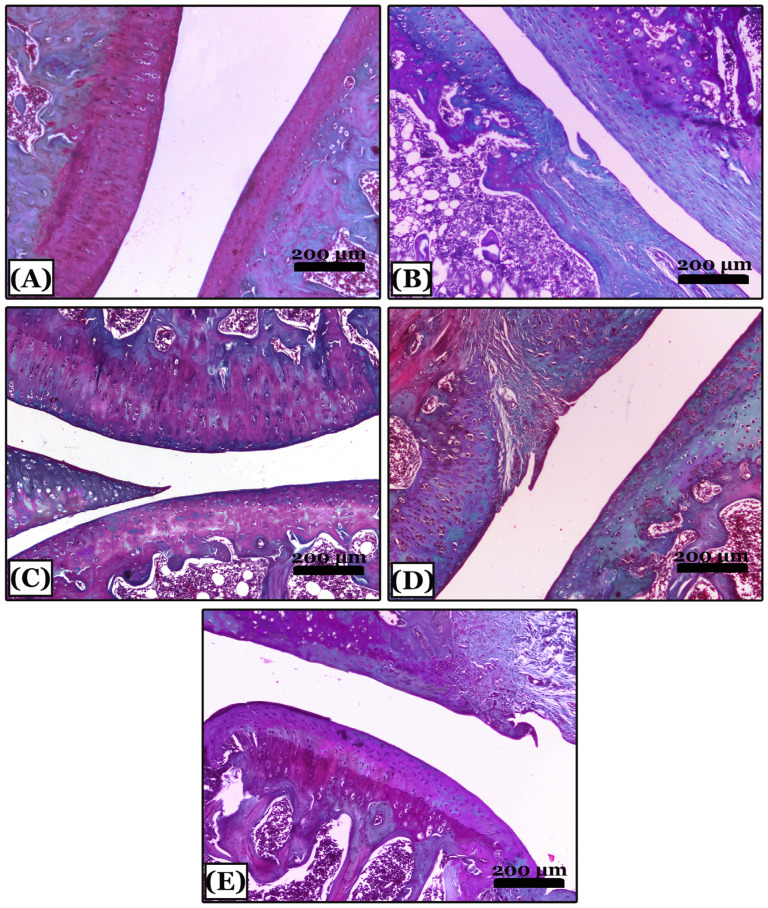
Histopathological evaluation of proteoglycans contents using Safranin-O-fast green stained sections in all studied groups. (**A**) note; the control negative group showing knee joints with a normal distribution of proteoglycans content of deep red color with intact cartilage. (**B**) note an osteoarthritic group with severe reduction and marked disappearance of proteoglycans content and articular cartilage fibrosis appeared with green color. (**C**) note; an indomethacin-treated group with minimal loss of proteoglycans content of red color in a few areas of articular cartilage. (**D**) note; the nano-naringenin-treated group with moderate loss of proteoglycans content and cartilage fibrosis in a small area. (**E**) note; an *Amphora coffeaeformis*-treated group showed uniform distribution and mild loss of proteoglycans content with intact cartilage. All photomicrographs ×100.

The control negative group showed normal proteoglycan content distribution and a uniform deep red color of the cartilaginous matrix with cartilage integrity in the SOFG-stained sections (Figure 5A). In contrast, the OA group showed in most areas, severe reduction and marked disappearance of proteoglycans content of the cartilaginous matrix and articular cartilage fibrosis (Figure 5B). The indomethacin-treated group revealed minimal loss of proteoglycans content in a few areas of the cartilaginous matrix with minimal cartilage surface degeneration (Figure 5C). The nano-naringenin-treated group showed moderate loss of proteoglycans content with a small area of cartilage degeneration (Figure 5D). Moreover, sections of *Amphorae coffeaeformis*-treated group revealed a uniform distribution with mild loss of proteoglycans content in the cartilaginous matrix with cartilage integrity (Figure 5E).

## 3. Discussion

MIA-induced osteoarthritis is one of the experimental OA models that have been demonstrated to have key clinical characteristics to animal osteoarthritis. It has been used to induce degenerative joint disease in the rat ankle [18]. Previous research has shown that infusion of MIA into the joint causes chondrocyte articular cartilage degeneration caused by inhibiting the activity of glyceraldehyde-3-phosphate dehydrogenase and, as a result, glycolysis inhibition. Chemically induced OA in rodents is similar to human OA, with functional loss and cartilage damage in the joints [19].

The primary goal of this experiment was to develop a therapy regimen for OA patients that could exhibit antioxidant and anti-inflammatory effects at the same time, based on the notion that both detrimental pro-inflammatory and pro-oxidant mediators participate in osteoarthritis.

The present results showed that the nano-naringenin and *Amphora coffeaeformis* significantly improved the body weights as compared to MIA-treated rat*s* (Table 1). This enhancement could be ascribed to the improved circumstances of the gastrointestinal system brought on by the antibacterial activity of both naringenin [20,21], and *Amphora coffeaeformis* [22]. *Amphora coffeaeformis* enhances growth performance and feed efficiency in Nile tilapia and broiler chickens [22,23]. The high level of polyunsaturated fatty acids (especially omega 3 FA), nutrients, antioxidants, and phenolic compounds in *Amphora coffeaeformis* could also be the reason.

The injection of MIA causes localized pain and inflammation in the rat joint indicated by the significantly larger right knee diameter and greater lameness grades observed on the 7th and 14th day post-MIA injection compared to the normal rats (Table 2 and Table 3). Our findings of the significantly decreased joint diameter and clinical score for lameness in rats treated with nano-naringenin and *Amphora coffeaeformis* suggest both anti-inflammatory and analgesic activity of these treatments that target the MIA-influenced joint inflammation and pain in rats, similar to indomethacin. The analgesic and anti-inflammatory properties of naringenin [24,25], *Amphora coffeaeformis* [16], and other microalgae [26] have been documented in earlier investigations.

The pathogenesis of OA has been linked to oxidative stress. Free radicals play important functions as secondary mediators in inflammation and can promote inflammatory hyperalgesia [27,28] and joint damage [29]. ROS is involved in transmitting a stimulatory signal to NF-κB; this triggers the expression of pro-inflammatory mediators [30].

According to our findings, MDA content in cartilage tissue significantly increased while GSH content significantly decreased in MIA-treated rats compared to control rats. (Table 4), revealing that an increase in pro-inflammatory mediators may cause lipid peroxidation in experimentally generated OA. According to multiple studies, human knee OA patients had considerably higher serum levels of enzymes connected to oxidative stress, MDA, and NO, than in control cases [31].

Our findings supported the antioxidant and anti-inflammatory effects of indomethacin, *Amphora coffeaeformis,* and nano-naringenin as indicated in the present study by the substantial reduction in MDA levels and the marked elevation in GSH levels in all treated groups compared with MIA-treated rats (Table 4). Hence, indomethacin, *Amphora coffeaeformis* followed by nano-naringenin seemed to be more potent in improving the oxidative stress in osteoarthritic rats. Previous reports also reported the antioxidant roles of nano-naringenin [32] and *Amphora coffeaeformis* [17,23].

Naringenin has a flavanone-like structure with three hydroxy groups at the 4’, 5’, and 7 carbons displaying more potent antioxidant activity [33]. In arthritic joints, naringenin may cause the mRNA expression of Nrf2 and its downstream effectors, hemoxygenase-1 (HO-1), and GSH, which inhibits hyperalgesia and provides tissue protection from oxidative stress damage [34].

Due to its high abundance of bioactive compounds such as flavonoids, carotenoids such as astaxanthin and canthaxanthin, and carotenoids such as astaxanthin and canthaxanthin, polyunsaturated fatty acids, β-glucans, and sulfated polysaccharides, vitamins C and E, and other polyphenolic antioxidants, *Amphora coffeaeformis* is regarded as a powerful radical scavenger [35,36]. These antioxidant compounds are functioning synergistically through their potential hydrogen-donating activity in vivo [37] and in vitro [38].

ADAMTSs and MMPs are metalloproteinases involved in cartilage ECM degradation and increased in osteoarthritis and rheumatoid arthritis joint tissues [39]. A member of the family of zinc-dependent matrix metalloproteinases (MMPs), MMP-3 is also referred to as stromelysin-1 [40]. MMP-3 is released by chondrocytes and synovial membrane cells in response to mechanical stimulation and pro-inflammatory cytokines [41]. MMP-3 levels in the blood could be used as a prospective disease indicator and a biomarker for osteoarthritis of the knee [42].

Previous studies revealed that the most active aggrecanases in human joint illness are ADAMTS-4 and ADAMTS-5 (aggrecanase-1 and aggrecanase-2, respectively), after analyzing synovial fluid samples from a variety of human joint diseases, including osteoarthritis [43,44]. According to the OA meniscus destabilization model, several researchers found that protecting mice’s joints against degeneration involved active ADAMTS-5 (aggrecanase 2), indicating that the ADAMTS-5-mediated breakdown of aggrecan is critical for the onset of arthritis. As a result, broad-spectrum metalloproteinase inhibitors have been developed as possible treatments [45].

TIMPs are membrane-bound proteins, such as metalloproteinases, that are secreted proteins that are localized at the cell surface. TIMP-3 is distinct in that it is confined to the ECM, most likely through attachment to the chondroitin sulfate and proteoglycans heparan sulfate. TIMPs 1–4 block all the active forms of MMPs; moreover, TIMPs are key regulators of cell surface elements as well as chemokine and cytokine functions, due to their pivotal function in metalloproteinase control [46]. TIMP-3 also inhibits the aggrecan degradation enzymes ADAMTS-1, ADAMTS-4, and ADAMTS-5 [47].

As a result, the detection of active MMP-3, ADAMTS-5, and TIMP-3 in clinical samples could reveal information regarding the development of arthritis and the response to therapy. According to our findings, rats with MIA-induced arthritis had marked elevation in serum levels of ADAMTS-5 and MMP-3 than did normal rats. In contrast, joint injury was indicated by a decreased TIMP-3 level in MIA rats compared to normal rats (Table 5). These outcomes matched previous results [39,48].

It would be beneficial if metalloproteinase medications could slow down matrix degeneration without impacting matrix synthesis. The current results showed that the treatment with indomethacin, *Amphora coffeaeformis* followed by nano-naringenin could induce a reduction in ADAMTS-5 and MMP-3 as well as an elevation in TIMP-3 (Table 5), suggestive of an anti-arthritic effect. Hence, the administration of these treatments seemed to be more potent in improving the anti-inflammatory markers and reduction of inflammatory joint markers.

Metalloproteinases are known to be inhibited by a variety of phytochemicals. Naringenin treatment decreased the expression of MMP-3 in MIA-induced arthritic rats [24] as well as in CFA-induced arthritic rats [32]. Previous studies had reported that some natural compounds such as polyphenol (epigallocatechin-3-gallate) [49], triterpenoids [50] and triptolite [51] have inhibitory activity against metalloproteinase production. Moreover, isoliquiritigenin inhibited NF-kB activation in chondrocytes and decreased IL-1β-induced synthesis of MMP-3, MMP-9, MMP-13, ADAMTS-4, and ADAMTS-5 [52]. Microalgae also contain anti-inflammatory have been demonstrated to reduce the production and activity of collagenases and aggrecanases as well as a variety of pro-inflammatory mediators in cartilage affected by osteoarthritis [53]. Therefore, the effect of *Amphora coffeaformis* may be related to high contents of anti-inflammatory linolenic acid (omega-3) and linoleic acid (omega-6) [54,55].

**Figure 6:**
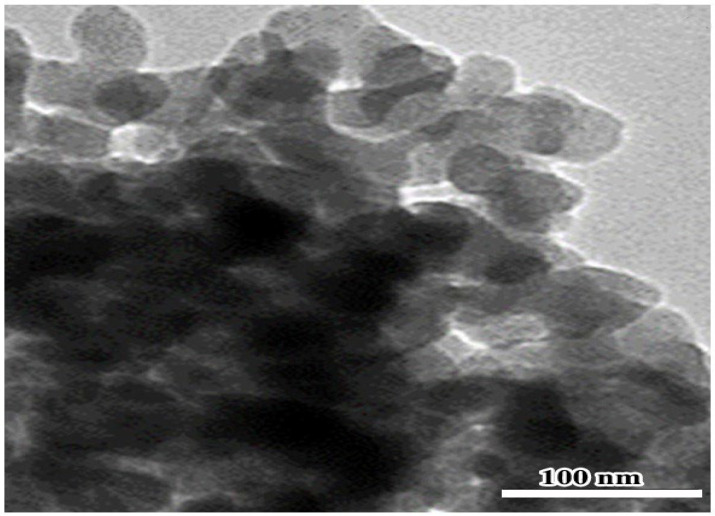
TEM pictures of nano-naringenin revealing the shape and size of the particles, which appear as spherical particles with an average size of 100 nm.

Because of the low oral bioavailability of naringenin, nanomaterial structures of sizes between 1 to 100 nm in at least one dimension can be utilized to raise the efficacy of drug delivery and their capacity to cross cell barriers to present an increased concentration [12]. In this study, TEM investigation revealed that the prepared nano-naringenin particles have a size of up to 100 nm with a spherical shape (Figure 6indicating greater nanoparticle uptake by cells. So, the potential effects of naringenin in a nano-size form are clearly detected.

All previously mentioned biochemical indices are compatible with our histopathological observations. In connection with these observations in the arthritic knee joint, there were chondrocyte apoptosis and multiple erosions in the articular cartilages thus replaced by collagen fibers and inflammatory cell infiltrations within the granulation tissue, synovitis including vascular congestion, edema, and inflammatory cell infiltrations, fibrosis, and multiple pannus formation of the synovial membrane. Moreover, articular bone erosions with osteoclast activity. That agrees with [56] who revealed that the inflammatory cell infiltrations play the main role in erosions of articular cartilages and bones in experimentally induced arthritis. Moreover, the aggregation of the inflammatory cells within the synovial fluid of the arthritic joint lead to pannus formation [57]. Thus, Kamarudin et al. [58] mentioned that the pannus formation instigates the erosion of the cartilage. The synoviocyte proliferation leads to synovial hyperplasia that was obtained due to the increase in macrophages and fibroblasts, which lead to a rise in cytokines production, such as TNF-α, IL-8, and IL-1β [59].

In accord with biochemical results, treatment with indomethacin significantly ameliorates all arthritic lesions in a large percentage by suppressing the inflammatory cell infiltrations through increasing anti-inflammatory cytokines and reduction of pro-inflammatory ones in comparison with the arthritic control. Our data revealed that treatment with nano-naringenin moderately retrieved some pathological arthritic lesions. There were only a few degenerative changes as well as inflammatory cell infiltrations and synovial hyperplasia. Additionally, data showed that treatment with *Amphorae coffeaeformis* markedly reduced most of the arthritic manifestations as the edematous swelling, synovial hyperplasia, pannus formation, cartilage damage, and the arthritic score with cartilage regeneration in some areas.

The findings indicate that Amphora coffeaeformis and nano-naringenin have anti-arthritic properties by lowering oxidative stress and inflammatory mediators in arthritis models brought on by MIA. It could be concluded that *Amphora coffeaeformis* followed by nano-naringenin are promising natural metalloproteinase inhibitors.

## 4. Materials and Methods

### 4.1. Chemicals

Naringenin (C15H12O5) (4′,5,7-trihydroxyflavanone, molecular weight of 272.26, 95%purity, CAS number: 67604-48-2) was purchased from *Glentham Life Sciences Ltd., Edinburgh, UK*. The *Amphorae coffeaeformis* powder was supplied by members of Algal Biotechnology Unit (National Research Centre, Dokki, Giza, Egypt). Malondialdehyde (MDA) and reduced glutathione (GSH) assaying commercial diagnostic kits were purchased from the Biodiagnostic Company for Research Kits in Egypt. ELISA kits for rat disintegrin and metalloproteinase with thrombospondin 5 repeats (ADAM TS-5) (catalog number: SEK205Ra), matrix metalloproteinase-3 (MMP-3) (catalog number: LS-F5516.) and rat tissue inhibitor of metalloproteinase-3 (TIMP-3) (catalog number: RK03988) were provided by R&D System, Minneapolis, MN, USA. Indomethacin, monosodium iodoacetate, and all other compounds with high analytical grades were supplied by Sigma Chemical Company (St. Louis, MO, USA).

### 4.2. Nano-Naringenin Preparation and Characterization

The approach of [60] was employed to manufacture nano-naringenin using a high-energy ball milling technique. At the Faculty of Postgraduate Studies for Advanced Sciences, Nanotechnology Lab, Beni-Suef University, nano-naringenin was created. The nano-naringenin was examined by a high-resolution TEM electron microscope at the National Research Center, Dokki, Giza, Egypt (Model: JEM-2100, JEOL Ltd., Tokyo, Japan). The nano-naringenin particles were approximately spherical crystals formed with homogeneous nanometric size spreading, according to the TEM (Figure 6).

### 4.3. Animals

Forty female Wister rats were classified into five equal groups. They were 7–9 weeks old and weighed 100–120 g. The animals were purchased from Al Nahda University, Beni-Suef, Egypt. The animals were kept under typical settings, which included ambient temperature of 20 to 25 °C, a regular daily lighting cycle of 10 to 12 h, a balanced standard feed, and unlimited access to water. Before the studies began, the animals were housed for two weeks to acclimate them to the environment.

### 4.4. Experimental Procedure

According to [61], while the animal was under anesthesia with ketamine (70 mg/kg) and xylazine (7 mg/kg), osteoarthritis was induced by administering a single intra-articular injection of 50 L physiological saline containing 2 mg monosodium iodoacetate (MIA 2 mg/50 L) into the articular space of the knee joint of the right hind leg. 

Five equally sized groups of eight rats each were classified as follow:

Group I: 50 μL isotonic sterile saline was injected into the right hind limb knee joint of normal control rats.

Group II: osteoarthritic rats (OA) were injected with MIA (2 mg/50 µL saline) as previously mentioned.

Group III: they were injected with MIA, and then treated with indomethacin (2 mg/kg) [62].

Group IV: they were injected with MIA, and then treated with nano-naringenin (25 mg/kg) [63].

Group V: they were injected with MIA, and then treated with *Amphorae coffeaeformis* (772 mg/kg) [14].

Medicaments were given once daily using an oral gavage tube initiated on the 8th day after osteoarthritis induction and continued for four weeks thereafter. The rats were monitored for clinical signs of osteoarthritis every week after MIA injection (on zero days till the end of experiment).

### 4.5. Joint Measurement (Evaluation of Knee Swelling and Degree of Lameness)

Using a calibrated digital caliper, the diameter of the right hind leg at the knee was assessed as a measure of joint edema and swelling rate in various groups. The measurements were taken weekly (on zero days till the end of experiment) after induction of osteoarthritis. Additionally, the body weight was measured weekly. The rats were monitored for detection of lameness weekly after MIA injection till the end of experiment. Using an ordinal scoring method, the degree of lameness in the control and OA groups was recorded. Based on subjective grading systems published by Khan et al. [64], the scoring system was created. Mild (01), less moderate (02), moderate (03), and severe were the grades for lameness (04).

### 4.6. Sampling and Tissue Preparations

At the end of experimental periods, under diethyl ether anesthesia, blood samples from the jugular vein and articular tissue specimens were collected from all animal groups. The blood samples were drawn into tubes, allowed to coagulate, and then centrifuged for 15 min at 3000 rpm. The clear supernatant sera were rapidly aspirated and stored at −20 °C until they were used for the investigation of several biochemical parameters relating to pro-inflammatory and anti-inflammatory joint markers. Knee joint tissue samples were quickly removed and separated into two portions after the decapitation and dissection. The first piece was kept for histological analysis in a 10% neutral buffered formalin solution. The second part was used for the preparation of 10% homogenate in 0.05 M phosphate buffer (pH 7) using a homogenizer (Ortoalresa, Spain). The homogenate was then centrifuged at 20,000 rpm for 20 min at 4 °C for removing the cell debris. 

### 4.7. Biochemical Analysis

Redox parameters including joint L-malondialdehyde (MDA) and reduced glutathione (GSH) content were detected as reported by [65,66], respectively. Whereas the serum levels of inflammatory (MMP-3, ADAMTS-5) and anti-inflammatory joint markers (TIMP-3) in the control and experimental groups were estimated by means of specific ELISA kits following the manufacturer’s instructions. ELISA is constructed on the idea of competitive binding [67]. All ELISA kits were measured by ELISA reader. Using an ELISA plate reader, color absorbance was measured at OD range of 490–630 nm (Stat Fax 2200, Awareness Technologies, Palm City, FL, USA).

### 4.8. Histopathological Analysis

By the end of our study, the right knee joint was dissected from rats of all studied groups, two knees were used for gross examination for OA and five trimmed knees were sunken in fixative (10% neutral buffered formalin) for 48 h, immersed for about three weeks in 20% EDTA solution until complete decalcification, then applying of the routine paraffin technique, and finally, 5 µm thickened sagittal sections stained by the following stains regarding [68].

(a) Hematoxylin and eosin stain (H&E) for the general screening. The degree scoring of the OA severity was assessed in joint sections stained with H&E X40 by OARSI system (Osteoarthritis Research Society International) as explained by [69,70,71] for pannus formation, synovitis, cartilage destruction, and bone erosion. The grades; are 0 (no change), 1 (minimal change), 2 (mild change), 3 (moderate change), and 4 (severe inflammation).
(b) Crossman’s Trichrome (CT) stain for collagen fibers identification and arthritis evaluation.
(c) Safranin O-Fast Green (SOFG) stain for proteoglycans demonstration and evaluation of the knee joints.

All stained slides were photographed with LEICA (DFC290 HD digital camera, St. Gallen, Switzerland).

### 4.9. Statistical Analysis

The statistical analysis was performed using SPSS v.25. Results were reported as mean ± standard error (SE) after using Duncan’s test post hoc for all statistical comparisons (SE). Values with a (*p* ≤ 0.05) were considered significant.

## 5. Conclusions

*Amphora coffeaeformis and* nano-naringenin have synergistic antioxidant and anti-inflammatory activities indicated in our study by the significant increase in joint GSH and serum TIMP-3 levels associated with a significant decrease in joint MDA, serum MMP-3, and ADMTS-5 levels, right knee diameter and lower clinical score for lameness in treated groups compared to MIA-arthritic rats, similarly as indomethacin. Therefore, *Amphora coffeaeformis and nano-*naringenin can be considered promising natural metalloproteinase inhibitors and should be taken into account when applied to food and medicine. Further clinical studies on their effects on human osteoarthritis should be applied.

## Figures and Tables

**Table 1:** Effect of nano-naringenin and *Amphora coffeaeformis* on body weight after induction of osteoarthritis.

Groups	Zero Day	1st Week	2nd Week	3rd Week	4th Week
Control negative	200.5 ± 5.45 ^a^	203.5 ± 2.93 ^a^	192.13 ± 2.63 ^ab^	210.88 ± 3.19 ^ab^	216.13 ± 6.02 ^a^
Osteoarthritic (OA)	193.25 ± 3.37 ^a^	189 ± 3.28 ^b^	185.63 ± 4.98 ^b^	192.5 ± 5.20 ^c^	197.75 ± 2.81 ^b^
OA + indomethacin	189.25 ± 2.17 ^a^	189.5 ± 3.74 ^b^	194.63 ± 3.6 ^ab^	196.63 ± 2.47 ^bc^	207.63 ± 4.35 ^ab^
OA + nano-naringenin	190.25 ± 2.12 ^a^	193.25 ± 3.56 ^b^	202.25 ± 4.99 ^a^	208.38 ± 4.70 ^ab^	220.25 ± 6.14 ^a^
OA + *Amphora coffeaeformis*	190.13 ± 5.81 ^a^	197.63 ± 2.22 ^ab^	203.38 ± 5.73 ^a^	213.88 ± 6.95 ^a^	220.63 ± 8.39 ^a^

Data expressed as (Mean ± standard error). The different superscript symbols (a, b, c) indicate that the means are significantly different.

**Table 2:** Effect of nano-naringenin and *Amphora coffeaeformis* on knee swelling.

Groups	Zero Day	1st Week	2nd Week	3rd Week	4th Week
Control negative	7.5 ± 0.19 ^a^	7.88 ± 0.30 ^b^	7.5 ± 0.19 ^b^	7.63 ± 0.18 ^b^	7.88 ± 0.30 ^c^
Osteoarthritic (OA)	7.5 ± 0.19 ^a^	18.13 ± 0.93 ^a^	15.5 ± 0.78 ^a^	11.63 ± 0.71 ^a^	10.5 ± 0.46 ^a^
OA + indomethacin	7.38 ± 0.18 ^a^	17.25 ± 0.70 ^a^	12.38 ± 0.50 ^ab^	9 ± 0.33 ^ab^	8.63 ± 0.26 ^b^
OA + nano-naringenin	7.63 ± 0.18 ^a^	17.38 ± 0.65 ^a^	13.63 ± 0.70 ^ab^	9.38 ± 0.42 ^ab^	9.13 ± 0.30 ^bc^
OA + *Amphora coffeaeformis*	7.5 ± 0.19 ^a^	17.13 ± 0.72 ^a^	13.37 ± 0.60 ^ab^	8.88 ± 0.30 ^ab^	8.63 ± 0.50 ^bc^

Data expressed as (Mean ± standard error). The different superscript symbols (a, b, c) indicate that the means are significantly different.

**Table 3:** Effect of nano-naringenin and *Amphora coffeaeformis* on clinical score of lameness at different weeks.

Groups	Zero Day	1st Week	2nd Week	3rd Week	4th Week
Control negative	0	0.38 ± 0.18 ^b^	0.38 ± 0.18 ^c^	0.25 ± 0.16 ^c^	0.13 ± 0.13 ^c^
Osteoarthritic (OA)	0	3.75 ± 0.16 ^a^	3.63 ± 0.18 ^a^	3.13 ± 0.30 ^a^	2.63 ± 0.18 ^a^
OA + indomethacin	0	3.63 ± 0.18 ^a^	2.88 ± 0.30 ^b^	2.13 ± 0.30 ^b^	0.75 ± 0.25 ^bc^
OA + nano-naringenin	0	3.63 ± 0.18 ^a^	3.25 ± 0.25 ^ab^	2.38 ± 0.18 ^b^	1.25 ± 0.25 ^b^
OA + *Amphora coffeaeformis*	0	3.63 ± 0.18 ^a^	3.00 ± 0.27 ^ab^	2.13 ± 0.30 ^b^	0.63 ± 0.26 ^bc^

Data expressed as (Mean ± standard error). The different superscript symbols (a, b, c) indicate that the means are significantly different.

**Table 4:** Effect of nano-naringenin and *Amphora coffeaeformis* on joint redox markers.

Groups	MDA (nmol/mg Tissue)	GSH (µg/mg Protein)
Control negative	0.61 ± 0.09 ^c^	1.61 ± 0.10 ^a^
Osteoarthritic (OA)	2.48 ± 0.05 ^a^	0.65 ± 0.06 ^c^
OA + indomethacin	0.68 ± 0.09 ^c^	1.69 ± 0.10 ^a^
OA + nano-naringenin	1.75 ± 0.05 ^b^	1.17 ± 0.04 ^b^
OA + *Amphora coffeaeformis*	0.73 ± 0.07 ^c^	1.65 ± 0.08 ^a^

Data expressed as (Mean ± standard error). The different superscript symbols (a, b, c) indicate that the means are significantly different.

**Table 5:** Effect of nano-naringenin and *Amphora coffeaeformis* on serum inflammatory/anti-inflammatory joint markers.

Groups	ADAM TS-5 (ng mL^−1^)	MMP-3 (ng mL^−1^)	TIMP-3 (ng mL^−1^)
Control negative	2.30 ± 0.12 ^d^	1.05 ± 0.1 ^c^	5.70 ± 0.32 ^a^
Osteoarthritic (OA)	10.70 ± 0.82 ^a^	3.17 ± 0.09 ^a^	1.97 ± 0.68 ^b^
OA + indomethacin	4.93 ± 0.30 ^c^	1.13 ± 0.09 ^c^	5.47 ± 1.08 ^a^
OA + nano-naringenin	8.93 ± 0.28 ^b^	1.63 ± 0.12 ^b^	3.43 ± 0.70 ^ab^
OA + *Amphora coffeaeformis*	6.07 ± 0.67 ^c^	1.13 ± 0.18 ^c^	5.50.64 ^a^

Data expressed as (Mean ± standard error). The different superscript symbols (a, b, c, d) indicate that the means are significantly different.

## Data Availability

Data is contained within the article.
